# Analysis of prognostic factors affecting immune checkpoint inhibitor therapy in tumor patients exposed to antibiotics

**DOI:** 10.3389/fonc.2023.1204248

**Published:** 2023-07-06

**Authors:** Qian Chen, Zhen Zhang, Xiaoli Li, Shaomei Feng, Shui Liu

**Affiliations:** ^1^ Department of Pharmacy, Beijing Gaobo Boren Hospital, Beijing, China; ^2^ Department of Adult Lymphoma, Beijing Gaobo Boren Hospital, Beijing, China; ^3^ Department of Pharmacy, Emergency General Hospital, Beijing, China

**Keywords:** antibiotics, immune checkpoint inhibitors, tumor, prognosis, screening, PFS, OS, meta-analysis

## Abstract

**Objective:**

Meta-analysis was performed to evaluate the prognostic factors in tumor patients treated with immune checkpoint inhibitors (ICIs) under antibiotic exposure.

**Method:**

Literature on the effect of antibiotics on the prognosis of tumor patients receiving ICIs was retrieved from Pubmed, Cochrane Library, EMbase, EBSCO Evidence-Based Medicine Database, China Biomedical Literature Database (CBM), and China National Knowledge Network (CNKI), and relevant influencing factors were extracted. Meta-analysis of efficacy was performed using RevMan 5.4 software.

**Results:**

A total of nine studies for 1,677 patients were included. The meta-analysis results showed that, in terms of progression-free survival, gender (male vs. female), Eastern Cooperative Oncology Group performance status (ECOG PS) (1–2 vs. 0), history of another cancer (yes vs. no), liver metastasis (yes vs. no), antibiotics (within the previous 2 months), PD-L1 (1%–49%), and PD-L1 (≥50%) factors are associated with progression-free survival in patients treated with ICIs under antibiotic exposure. In terms of overall survival, gender (male vs. female), ECOG score (1–2 vs. 0), history of another cancer (yes vs. no), brain metastasis (yes vs. no), liver metastasis (yes vs. no), radiation (within the previous 3 months), antibiotics (within the previous 2 months), PD-L1 (1%–49%), and PD-L1 (≥50%) factors are associated with overall survival in patients with antibiotic exposure receiving ICIs for tumor treatment.

**Conclusion:**

Gender, ECOG score, history of another cancer, brain metastasis, liver metastasis, radiation (within the previous 3 months), antibiotics (within the previous 2 months), PD-L1 (1%–49%), and PD-L1 (≥50%) were associated with clinical benefit in patients with antibiotic exposure receiving ICIs for tumor treatment. Based on the above-mentioned factors, clinicians can screen cancer patients who receive ICIs under antibiotic exposure and rationally use antibiotics and ICIs in combination.

## Introduction

Tumor disease is one of the common research objects in clinical research at present. All kinds of solid tumors have a high incidence. The clinical treatment for tumor mainly includes drug therapy and surgical therapy (1). Among all therapeutic strategies, immunotherapy is one of the most promising ones. In terms of existing clinical evidence, the use of immune checkpoint inhibitors has provided help for the treatment of tumor patients, especially for improving overall survival, disease-free progression, and other clinical benefits of tumor patients (2). In recent years, the role of intestinal microflora in the treatment of immune checkpoint inhibitors has attracted the attention of researchers, and the use of antibiotics can greatly affect the intestinal microflora in a relatively short period of time (3). However, in clinical practice, many patients will choose to use antibiotics simultaneously in the treatment process, which will affect the clinical benefits of patients and is not conducive to the treatment and prognosis of patients (4). Numerous retrospective studies and meta-analyses (5, 6) have shown that antibiotic exposure is associated with poor prognosis in patients receiving immune checkpoint inhibitors (ICIs) treatment in various malignant tumors. Similarly, similar results were found in studies of other non-small cell lung carcinoma (NSCLC) patients receiving ICIs treatment. In a retrospective study of 157 patients with metastatic NSCLC who received ICIs treatment (7), compared with patients who did not receive antibiotics, the overall survival (5.1 vs. 13.2 months, *P* < 0.0001) and progression-free survival (PFS; 1.9 vs. 3.5 months, *P* < 0.0001) of patients receiving antibiotic treatment were significantly shortened. All previous similar retrospective studies (8) analyzed the impact of antibiotics on the outcome of ICIs treatment, but these studies were based on single-arm studies without a control group or patient cohorts treated in a single institution. In addition, similar results were found in a large observational study (9), which included advanced NSCLC patients receiving first-line pembrolizumab monotherapy and compared with the cohort of patients receiving standard chemotherapy. In the population receiving pembrolizumab treatment, the overall survival (OS; 10.4 vs. 17.2 months) and PFS (4.8 vs. 7.5 months) of patients in the antibiotic group were significantly shorter than those in the non-antibiotic group, However, no similar differences were observed in patients receiving chemotherapy. For patients who must use antibiotics to control infection, clinicians need to predict, based on patient biomarkers, the factors that affect the clinical outcome of antibiotic exposure to immune checkpoint inhibitors. Therefore, the integration of multiple clinical and molecular features is necessary to accurately predict the clinical outcome of tumors treated with immune checkpoint inhibitors under antibiotic exposure. In this study, meta-analysis was used to systematically evaluate the prognostic factors of tumor patients treated with immune checkpoint inhibitors under antibiotic exposure in order to judge and analyze the influence of clinical immunotherapy effects.

## Materials and methods

### Content of the study

This study intends to search the relevant domestic and foreign literature and to include randomized controlled trials or clinical trials of immune checkpoint inhibitors in the treatment of cancer patients with antibiotic exposure. The patients were grouped according to age (<65 vs. ≥65), gender (male vs. female), Eastern Cooperative Oncology Group performance status (ECOG PS; 1–2 vs. 0), smoking status (smoking vs. non-smoking), PD-L1 expression level (1%–49%, ≥50%), history of another cancer (yes vs. no), brain metastasis (yes vs. no), liver metastasis (yes vs. no), radiation (yes vs. no), BRAF mutation status (yes vs. no), history of antibiotic use (yes vs. no), and combination of PD-1 inhibitors. We conducted a meta-analysis to systematically evaluate the clinical and molecular characteristics of the immune checkpoint inhibitor treatment response in cancer patients exposed to antibiotics in order to provide evidence for clinical decision-making.

### Search strategy

According to the research topic, following the Participants, Intervention, Control, Outcome, Study design (PICOS) principle, the relevant subject words and free words were selected, and the retrieval scheme was formulated. The retrieval process was followed step by step. Two researchers independently searched Pubmed, Cochrane Library, EMbase, EBSCO Evidence-Based Medicine Database, China Biology Medicine Disc (CBM), and China National Knowledge Infrastructure (CNKI) for relevant studies on the effect of antibiotics on the efficacy of immune checkpoint inhibitors in cancer. The search time limit was from the establishment of the database to January 2023. In addition, the references of the included literature were traced back to supplement the acquisition of relevant literature. Additional studies were retrieved from the proceedings of the American Association for Cancer Research (AACR), Chinese Society of Clinical Oncology (CSCO), American Society of Clinical Oncology (ASCO), China Anti-cancer Association (CACA), and European Society for Medical Oncology (ESMO) and other meetings to include more complete data. The search adopts the combination of subject words and free words mainly including antibiotics, tumors, immunotherapy, immune checkpoint inhibitors, PD-1/PD-L1 inhibitors, nivolumab, pembrolizumab, atezolizumab, etc.

### Study selection

#### Inclusion criteria

Data from clinical trials, randomized controlled trials (RCTs), or retrospective studies on cancer patients treated with immune checkpoint inhibitors under antibiotic exposure were collected. Eligible patients were adults with a pathologically confirmed diagnosis of cancer who received antibiotics before, during, or after treatment with an immune checkpoint inhibitor. The outcome measures were the hazard ratio (HR) and 95% confidence interval (CI) of PFS and/or OS in patients with age (<65 vs. ≥65), gender (male vs. female), ECOG PS (1–2 vs. 0), smoking status (smoking vs. non-smoking), PD-L1 expression level (1%–49%, ≥50%), history of another cancer (yes vs. no), brain metastasis (yes vs. no), liver metastasis (yes vs. no), radiation (yes vs. no), BRAF mutation status (yes vs. no), antibiotic use history (within the previous 2 months), and co-treatment with PD-1 drugs.

#### Exclusion criteria

(1) Articles without explicit mention of randomization
(2) Repeated publications
(3) Literature types that were reviews, cell and animal experimental studies, case reports, etc.
(4) Incomplete data could not be extracted
(5) Unclear diagnostic criteria
(6) Literature other than Chinese or English

### Literature screening and data extraction

Two reviewers independently screened the literature and extracted and cross-checked the data. In case of disagreement, a third party was consulted to assist in judgment, and in case of lacking data, the authors were tried to be contacted to supplement the needed information. The titles and abstracts were first read during literature screening, and after excluding clearly irrelevant literature, the full text was further read to determine the final inclusion. The hazard ratios and 95% confidence intervals for PFS and/or OS were according to the following characteristics: age (<65 vs. ≥65), gender (male vs. female), ECOG PS (1–2 vs. 0), BRAF mutation status (yes vs. no), smoking status (smoking vs. non-smoking), PD-L1 expression level (1%–49%, ≥50%), previous cancer history (yes vs. no), brain metastasis (yes vs. no), liver metastasis (yes vs. no), radiation (yes vs. no), antibiotic use history, and co-treatment with PD-1 inhibitors.

### Quality assessment of the included literature

The researchers evaluated the quality of the literature in strict accordance with the bias risk plan recommended by the Cochrane Network (10). Review Manager 5.4 software was used to evaluate the content of the included RCTs, including the following: (1) random sequence generation, (2) allocation concealment, (3) blinding of participants and personnel, (4) blinding of outcome assessment, (5) incomplete outcome data, (6) selective reporting, and (7) other biases.

### Statistical analysis

The methods described in the Cochrane Handbook for meta-analysis were used to analyze the data using Review Manager 5.4 statistical software. Pooled estimates of PFS and OS were expressed as HRs, 95% CIs, and *P*-values calculated by the inverse variance weighting method. Each model is analyzed separately in a univariate model. The *P*-value of the forest plot in the meta-analysis was used to detect the difference between trials, and the results were considered significantly different when the *P*-value was <0.05. Chi-square test was used to test the heterogeneity of the included studies: if the heterogeneity was small (*P* > 0.1 and *I* ^2^ ≤ 50%), fixed-effect model was used for meta-analysis. If the heterogeneity was large (*P* ≤ 0.1 and *I* ^2^ >50%), random-effects model was used. If the data provided by the trial could not be subjected to meta-analysis, only a descriptive qualitative analysis was performed. According to the Cochrane Handbook for Systematic Reviews, publication bias was tested by funnel plots. In addition, there are multiple influencing factors and endpoints in the results of this study, all of which are univariate analyses, and there is no mutual influence between each endpoint, so there is no need for multiple adjustments.

## Results

### Literature screening process

**Figure 1:**
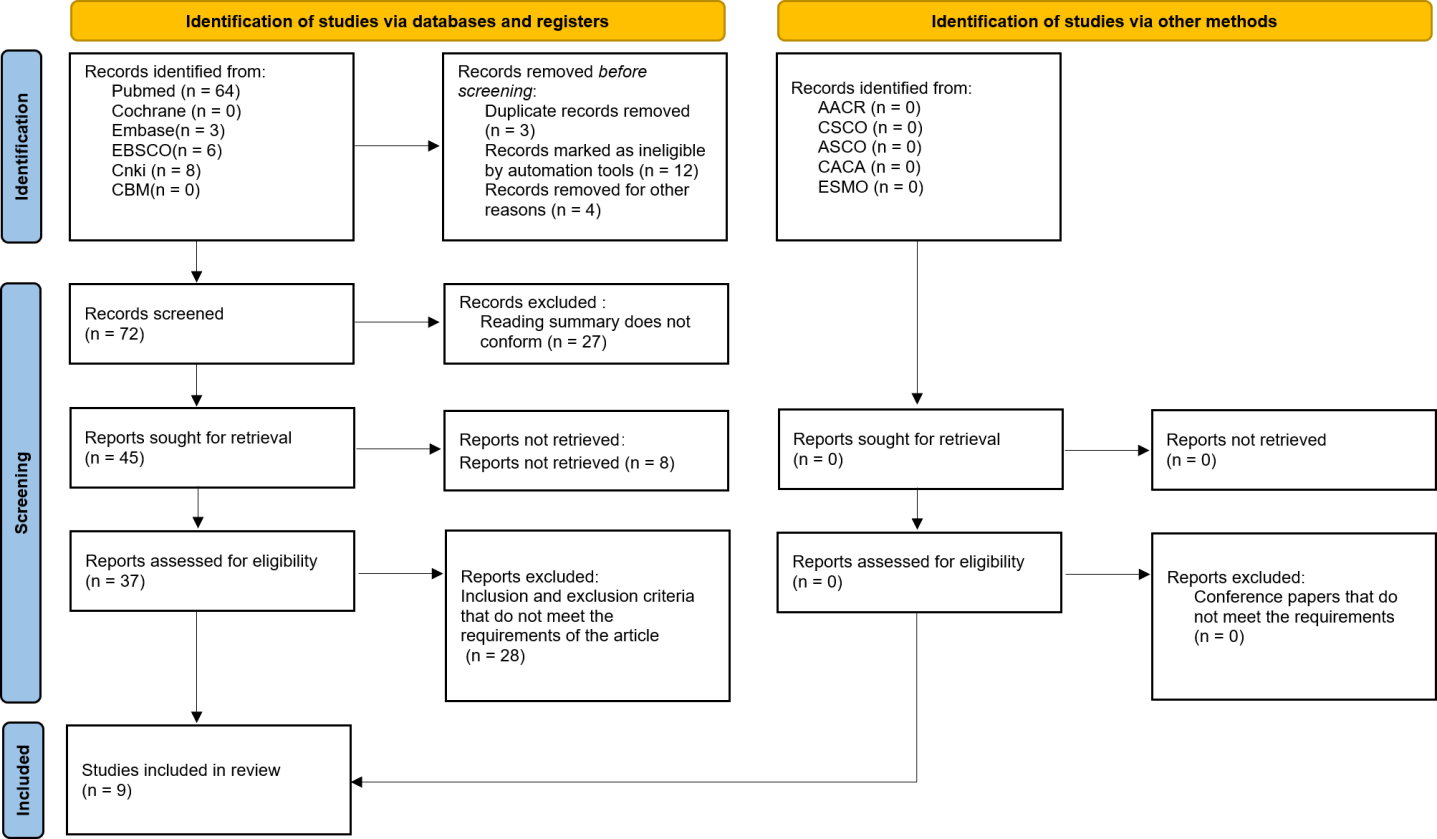
PRISMA flow chart of article selection.

A total of 81 relevant literatures were obtained through database retrieval, including eight Chinese literatures, 73 English literatures, and 23 conference papers and abstracts. Moreover, 67 duplicates, case reports, reviews, and irrelevant contents were excluded. In addition, 37 literatures were screened strictly according to the above-mentioned screening process, and nine (11–19) studies were finally included for the quantitative analysis, as shown in **Figure 1**.

### Data characteristics included in the study and results of bias risk assessment

**Figure 2:**
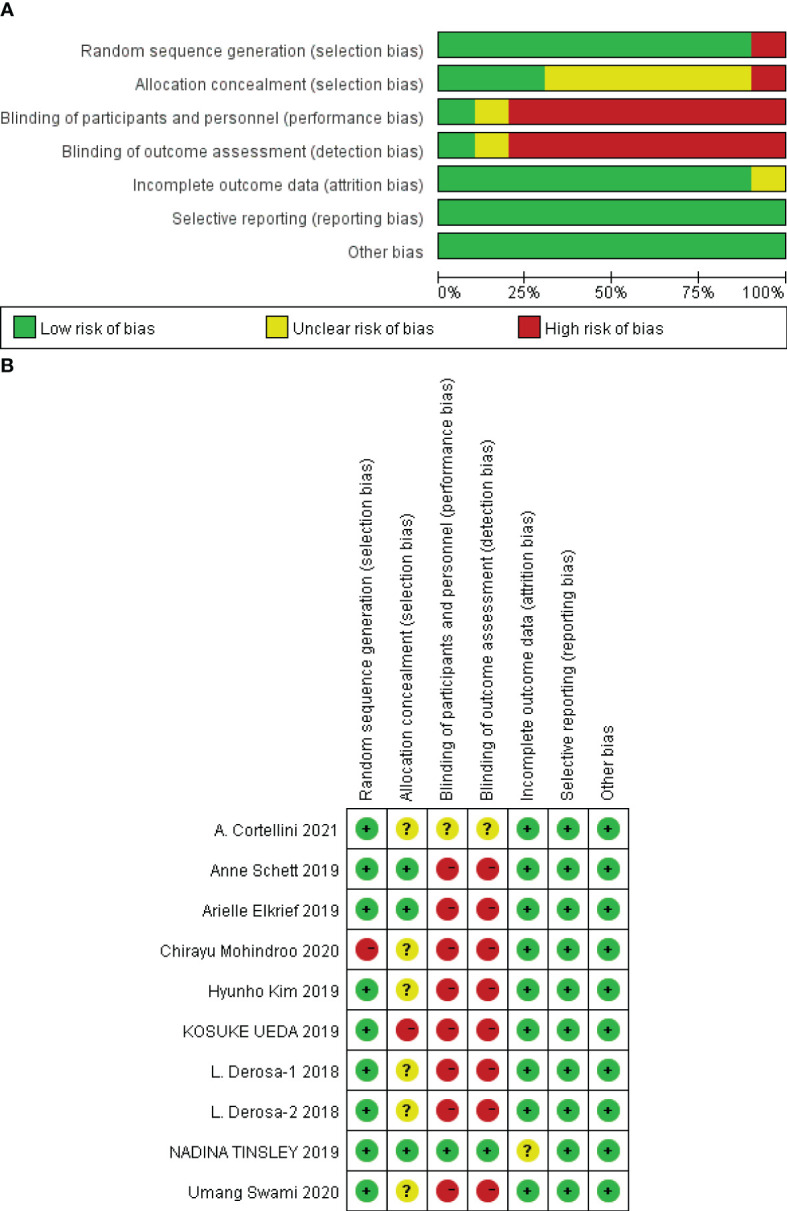
**(A, B)** Evaluation results of the methodology quality of the included studies.

A total of 1,677 eligible cancer patients were included in the 19 studies, and the general characteristics are shown in **Tables 1**, **2**. All nine studies were randomized controlled trials, and the quality of the literature was evaluated by the bias risk summary chart in Review Manager 5.4. The results of the risk of bias assessment are shown in **Figures 2A, B**.

Table 1Multivariate analysis for predictors of progression-free survival, HR (95%CI), *p*-value.

**Table:** 

Author	Year	Sample size (F/M)	Gender (male vs. female)	Smoking status (smoking vs. non-smoking)	ECOG PS (1–2 vs. 0)	History of another cancer (yes vs. no)	Brain metastasis (yes vs. no)	Liver metastasis (yes vs. no)	BRAF mutation (yes vs. no)
Umang Swami (11)	2020	59/110	1.09 [0.75, 1.60] *p* = 0.65	1.03 [0.60, 1.77] *p* = 0.80	NA	0.90 [0.42, 1.93] *p* = 0.78	1.84 [1.22, 2.76] *p* < 0.01	1.47 [0.91, 2.36] *p* = 0.11	0.84 [0.58, 1.22] *p* = 0.36
A. Cortellini (12)	2021	115/187	1.03 [0.77, 1.38] *p* = 0.7994	1.27 [0.70, 2.30] *p* = 0.4139	2.06 [1.35, 3.12] *p* = 0.0007	NA	1.04 [0.75, 1.44] *p* = 0.7785	1.16 [0.78, 1.74] *p* = 0.0454	NA
KOSUKE UEDA (13)	2019	24/7	1.941 [0.67, 5.12] *p* = 0.2106	NA	NA	NA	NA	0.842 [0.19, 2.58] *p* = 0.7830	NA
Anne Schett (14)	2019	87/131	1.06 [0.62, 1.82] *p* = 0.82	0.42 [0.17, 1.03] *p* = 0.06	0.90 [0.51, 1.58] *p* = 0.70	1.66 [0.86, 3.20] *p* = 0.13	1.04 [0.75, 1.44] *p* = 0.29	NA	NA
Chirayu Mohindroo (15)	2020	147/195	0.83 [0.65, 1.07] *p* = 0.15	NA	NA	NA	NA	NA	NA
Arielle Elkrief (16)	2019	34/40	0.95 [0.50, 179] *p* = 0.87	NA	0.42 [0.24, 0.77] *p* = 0.01	NA	NA	NA	0.94 [0.38, 2.35] *p* = 0.90
L. Derosa-1 (17)	2018	41/80	NA	NA	NA	2.1 [1.3, 3.3] *p* < 0.01	NA	NA	NA
L. Derosa-2 (17)	2018	41/80	NA	0.7 [0.5, 1.0] *p* = 0.04	1.7 [1.2, 2.4] *p* < 0.01	1.1 [0.8, 1.5] *p* = 0.92	NA	NA	NA
NADINA TINSLEY (18)	2019	110/181	1.3 *p* = 0.07	NA	1.419 *p* = 0.028	1.42 *p* = 0.016	1.276 *p* = 0.233	NA	NA
Hyunho Kim (19)	2019	66/168	NA	NA	2.316 [1.128, 4.187] *p* = 0.005	1.064 [0.70, 1.61] *p* = 0.768	0.83 [0.48, 1.42] *p* = 0.502	NA	NA

**Table:** 

Author	Prior systemic therapy (yes vs. no)	Radiation (within the previous 3 months)	Antibiotics (within the previous 2 months)	Age (years) (<65 vs. ≥65)	PD-L1 (1%–49%)	PD-L1 (>50%)	Anti-PD(L)1 compound—nivolumab	Anti-PD(L)1 compound—pembrolizumab	Anti-PD(L)1 compound—atezolizumab
Umang Swami (11)	0.94 [0.64, 1.36] *p* = 0.73	1.60 [1.00, 2.58] *p* = 0.05	1.28 [0.80, 2.04] *p* = 0.30	1.05 [0.93, 1.17] *p* = 0.46	NA	NA	NA	NA	NA
A. Cortellini (12)	NA	NA	1.12 [0.76, 1.63] *p* = 0.5552	NA	0.80 [0.57, 1.12] *p* = 0.2029	0.55 [0.37, 0.81] *p* = 0.0024	NA	NA	NA
KOSUKE UEDA (13)	NA	NA	6.518 [1.857, 21.416] *p* = 0.0048	1.21 [0.46, 3.34] *p* = 0.6981	NA	NA	NA	NA	NA
Anne Schett (14)	NA	1.13 [0.50, 2.54] *p* = 0.78	3.45 [1.44, 8.29] *p* < 0.01	0.68 [0.41, 1.14] *p* = 0.14	7.06 [2.55, 19.5] *p* < 0.01	4.24 [1.66, 10.8] *p* < 0.01	3.86 [2.28, 6.53] *p* < 0.01	1.71 [0.81, 3.62] *p* = 0.16	1.36 [0.17, 10.6] *p* = 0.77
Chirayu Mohindroo (15)	NA	NA	0.95 [0.74, 1.22] *p* = 0.68	1.04 [0.82, 1.33] *p* = 0.73	NA	NA	NA	NA	NA
Arielle Elkrief (16)	NA	NA	0.32 [0.13, 0.83] *p* = 0.02	0.93 [0.52, 1.63] *p* = 0.79	NA	NA	NA	NA	NA
L. Derosa-1 (17)	NA	NA	2.20 [1.3, 3.3] *p* = 0.02	1.2 [0.8, 1.8] *p* = 0.43	NA	NA	NA	NA	NA
L. Derosa-2 (17)	1.4 [1.0, 1.9] *p* = 0.05	NA	1.4 [1.0, 2.0] *p* = 0.04	1.2 [0.9, 1.6] *p* = 0.2	NA	NA	NA	NA	NA
NADINA TINSLEY (18)	0.880 *p* = 0.436	NA	1.564 *p* = 0.003	1.001 *p* = 0.940	NA	NA	NA	NA	NA
Hyunho Kim (19)	NA	NA	1.948 [1.31, 2.89] *p* = 0.001	NA	1.723 [0.78, 3.85] *p* = 0.181	1.165 [0.55, 2.49] *p* = 0.693	1.001 *p* = 0.891	1.115 [0.72, 1.74] *p* = 0.631	1.043 [0.59, 1.83] *p* = 0.884

F, female; M, male; HR, hazard ratio; NA, not available; ECOG PS, Eastern Cooperative Oncology Group performance status.

Table 2Multivariate analysis for the predictors of overall survival, HR [95%CI], *p*-value.

**Table:** 

Author	Year	Sample size (F/M)	Gender (male vs. female)	Smoking status (smoking vs. non-smoking)	ECOG PS (1–2 vs. 0)	History of another cancer (yes vs. no)	Brain metastasis (yes vs. no)	Liver metastasis (yes vs. no)	Prior systemic therapy (yes vs. no)
Umang Swami (11)	2020	59/110	0.98 [0.60, 1.59] *p* = 0.93	1.25 [0.66, 2.36] *p* = 0.79	NA	0.57 [0.18, 1.82] *p* = 0.35	3.41 [2.13, 5.46] *p* < 0.01	2.06 [1.22, 3.48] *p* < 0.01	1.14 [0.72, 1.82] *p* = 0.57
A. Cortellini (12)	2021	115/187	1.19 [0.83, 1.69] *p* = 0.3273	1.81 [0.86, 3.79] *p* = 0.1128	3.01 [1.88, 4.83] *p* < 0.0001	NA	1.30 [0.88, 1.91] *p* = 0.1856	1.46 [0.93, 2.30] *p* = 0.0974	NA
Anne Schett (14)	2019	87/131	1.83 [0.85, 3.91] *p* = 0.12	0.44 [0.15, 1.28] *p* = 0.13	0.58 [0.29, 1.17] *p* = 0.13	2.52 [1.14, 5.58] *p* = 0.02	1.30 [0.60, 2.83] *p* = 0.51	NA	NA
Chirayu Mohindroo (15)	2020	147/195	0.82 [0.64, 1.06] *p* = 0.13	NA	NA	NA	NA	NA	NA
L. Derosa-1 (17)	2018	41/80	NA	NA	NA	2.4 [1.2, 4.6] *p* < 0.01	NA	NA	NA
L. Derosa-2 (17)	2018	41/80	NA	1.2 [0.7, 1.9] *p* = 0.55	3.6 [1.9, 6.5] *p* < 0.01	1.4 [0.8, 1.6] *p* = 0.57	NA	NA	1.9 [1.3, 2.9] *p* < 0.01
NADINA TINSLEY (18)	2019	110/181	1.126 *p* = 0.495	NA	1.647 *p* = 0.01	1.526 *p* = 0.018	1.373 *p* = 0.263	NA	0.950 *p* = 0.788
Hyunho Kim (19)	2019	66/168	NA	NA	2.46 [1.29, 4.71] *p* = 0.006	0.956 [0.59, 1.54] *p* = 0.855	1.008 [0.55, 1.84] *p* = 0.979	NA	NA

**Table:** 

Author	Radiation (within the previous 3 months)	Antibiotics (within the previous 2 months)	Age (years) (<65 vs. ≥65)	PD-L1 (1%–49%)	PD-L1 (>50%)	Anti-PD(L)1 compound—nivolumab	Anti-PD(L)1 compound—pembrolizumab	Anti-PD(L)1 compound—atezolizumab	
Umang Swami (11)	2.35 [1.36, 4.06] *p* < 0.01	1.73 [1.00, 2.99] *p* = 0.05	1.11 [0.95, 1.29] *p* = 0.19	NA	NA	NA	NA	NA	
A. Cortellini (12)	NA	1.42 [0.91, 2.22] *p* = 0.1207	NA	0.93 [0.61, 1.43] *p* = 0.7731	0.59 [0.37, 0.94] *p* = 0.0282	NA	NA	NA	
Anne Schett (14)	1.60 [0.56, 4.51] *p* = 0.38	3.71 [1.34, 10.4] *p* = 0.01	0.98 [0.51, 1.90] *p* = 0.96	7.08 [2.60, 19.3] *p* < 0.01	3.29 [1.13, 9.58] *p* = 0.03	4.29 [2.48, 7.39] *p* < 0.01	2.12 [0.88, 5.08] *p* = 0.09	1.09 [0.14, 18.47] *p* = 0.93	
Chirayu Mohindroo (15)	NA	0.99 [0.76, 1.28] *p* = 0.93	0.94 [0.73, 1.21] *p* = 0.64	NA	NA	NA	NA	NA	
L. Derosa-1 (17)	NA	2.4 [1.1, 5.7] *p* = 0.04	0.8 [0.4, 1.5] *p* = 0.43	NA	NA	NA	NA	NA	
L. Derosa-2 (17)	NA	2.9 [1.9, 4.4] *p* < 0.01	1.3 [0.9, 1.9] *p* = 0.23	NA	NA	NA	NA	NA	
NADINA TINSLEY (18)	NA	1.699 *p* = 0.002	1.012 *p* = 0.076	NA	NA	NA	NA	NA	
Hyunho Kim (19)	NA	2.476 [1.568, 3.911] *p* < 0.001	NA	1.839 [0.67, 4.99] *p* = 0.233	1.329 [0.52, 3.43] *p* = 0.557	1.001, *p* = 0.363	1.31 [0.79, 2.173] *p* = 0.295	0.801 [0.410, 1.564] *p* = 0.516	

F, female; M, male; HR, hazard ratio; NA, not available; ECOG PS, Eastern Cooperative Oncology Group performance status.

### Meta-analysis results

#### Meta-analysis of the influencing factors of PFS

**Figure 3:**
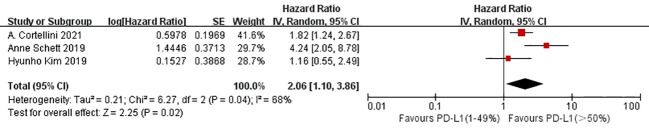
Meta-analysis results of PD-L1 (>50%) in progression-free survival.

**Figure 4:**
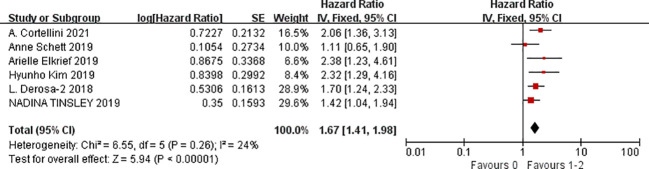
Meta-analysis results of ECOG (1–2 vs. 0) in progression-free survival.

**Figure 5:**
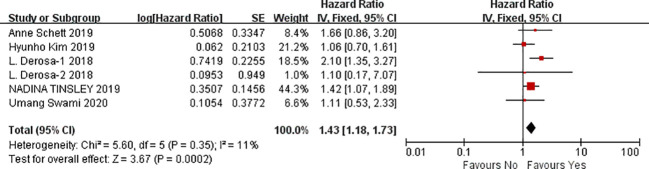
Meta-analysis results of the history of another cancer in progression-free survival.

A total of nine studies were available to analyze the influencing factors of progression-free survival in cancer patients treated with ICIs under antibiotic exposure. The outcome indicators mainly included gender (male vs. female), age (<65 vs. ≥65), smoking status (smoking vs. non-smoking), ECOG score (1–2 vs. 0), previous cancer history (yes vs. no), brain metastasis (yes vs. no), liver metastasis (yes vs. no), BRAF mutation status (yes vs. no), previous systemic therapy (yes vs. no), radiation in the past 3 months, antibiotic use in the past two months, PD-L1 expression level (1%–49%, ≥50%), and combined PD-1 drugs (nivolumab, pembrolizumab, and atezolizumab). The results of the meta-analysis showed that under the influencing factors of gender (male vs. female) (HR = 1.14, 95%CI = 1.01–1.30, *P* = 0.04), ECOG PS (1–2 vs. 0) (HR = 1.67, 95%CI = 1.41–1.98, *P* < 0.0001), history of another cancer (yes vs. no) (HR = 1.43, 95%CI = 1.18–1.73, *P* = 0.0002), liver metastasis (yes vs. no) (HR = 1.18, 95%CI = 1.03–1.36, *P* = 0.02), antibiotics (within the previous 2 months) (HR = 1.42, 95%CI = 1.25–1.62, *P* < 0.0001), PD-L1 (1%–49%) (HR = 1.06, 95%CI = 1.03–1.09, *P* < 0.0001), and PD-L1 (≥50%) (HR = 2.06, 95%CI = 1.10–3.86, *P* = 0.02), the treatment of tumor patients with ICIs under antibiotic exposure led to a significant reduction in PFS. In cancer patients with PD-L1 expression ≥50%, the use of antibiotics during ICIs treatment was 2.06 times more effective in shortening the time without disease progression than the no-antibiotic treatment. Furthermore, ECOG PS1–2 score, history of another cancer (yes vs. no), and antibiotics (within the previous 2 months) had a greater impact on the shortening of progression-free period caused by using antibiotics during ICIs which was 1.67 times, 1.43 times, and 1.42 times of those without antibiotic treatment, respectively. Such patients should be screened in clinical treatment, and their medical history should be asked in detail to avoid the use of antibiotics in immunotherapy. However, age (<65 vs. ≥65), smoking status (smoking vs. non-smoking), brain metastasis (yes vs. no), BRAF mutation status (yes vs. no), previous systemic therapy (yes vs. no), radiation exposure within the past 3 months, and combined PD-1 inhibitors (nivolumab, pembrolizumab, and atezolizumab) were not associated with PFS, and the results were not statistically significant, as shown in **Figures 3**–**5** (due to the large number of figures, only the top three influencing factors are reflected) and **Table 3**.

**Table 3:** Meta-analysis results of predictors of progression-free survival.

Outcomes	Studies	Heterogeneity test results		Effect model	Meta-analysis results	
		*P*	*I^2^*		95%CI	*P*
Gender (male vs. female)	7	0.81	0%	Fixed	1.14 [1.01, 1.30]	0.04
Age (<65 vs. ≥65)	8	0.65	0%	Fixed	1.01 [0.98, 1.03]	0.61
Smoking status (smoking vs. non-smoking)	4	0.18	39%	Fixed	1.18 [0.99, 1.41]	0.06
ECOG PS (1–2 vs. 0)	6	0.26	24%	Fixed	1.67 [1.41, 1.98]	<0.0001
History of another cancer (yes vs. no)	6	0.35	11%	Fixed	1.43 [1.18, 1.73]	0.0002
Brain metastasis (yes vs. no)	5	0.04	60%	Random	1.20 [1.98, 1.47]	0.08
Liver metastasis (yes vs. no)	3	0.64	0%	Fixed	1.18 [1.03, 1.36]	0.02
BRAF mutation (yes vs. no)	2	0.83	0%	Fixed	1.17 [0.83, 1.66]	0.37
Prior systemic therapy (yes vs. no)	3	0.51	0%	Fixed	1.19 [0.98, 1.45]	0.07
Radiation (within the previous 3 months)	2	0.49	0%	Fixed	1.48 [0.98, 2.23]	0.06
Antibiotics (within the previous 2 months)	10	0.003	65%	Random	1. 42 [1.25, 1.62]	<0.0001
PD-L1 (1%–49%)	3	0.32	13%	Fixed	1.06 [1.03, 1.09]	<0.0001
PD-L1 (≥50%)	3	0.04	68%	Random	2.06 [1.10, 3.86]	0.02
Anti-PD(L)1 compound—nivolumab	2	0.0001	93%	Random	1.88 [1.50, 7.04]	0.35
Anti-PD(L)1 compound—pembrolizumab	2	0.34	0%	Fixed	1.25 [0.85, 1.83]	0.26
Anti-PD(L)1 compound—atezolizumab	2	0.81	0%	Fixed	1.06 [0.62, 1.83]	0.83

ECOG PS, Eastern Cooperative Oncology Group performance status.

#### Meta-analysis of the influencing factors of OS

**Figure 6:**
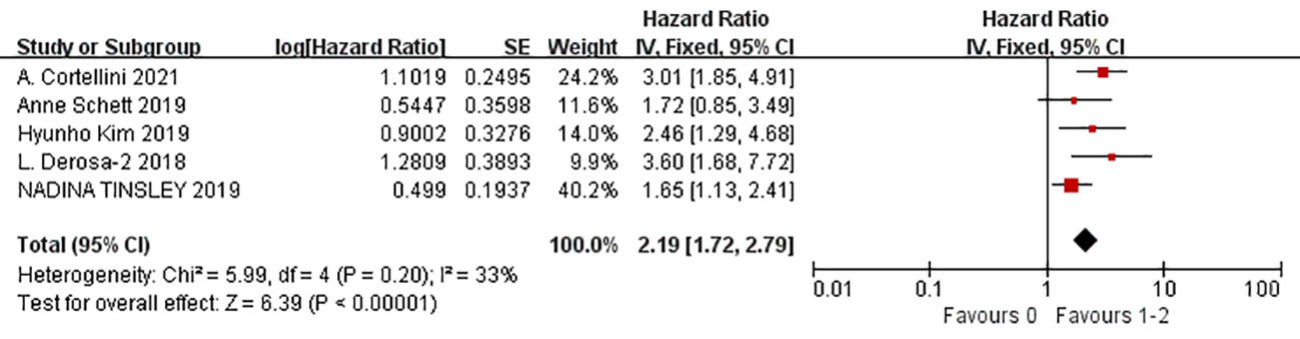
Meta-analysis results of ECOG (1–2 vs. 0) in overall survival.

**Figure 7:**
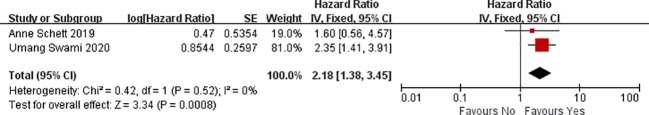
Meta-analysis results of radiation (within the previous 3 months) in overall survival.

**Figure 8:**
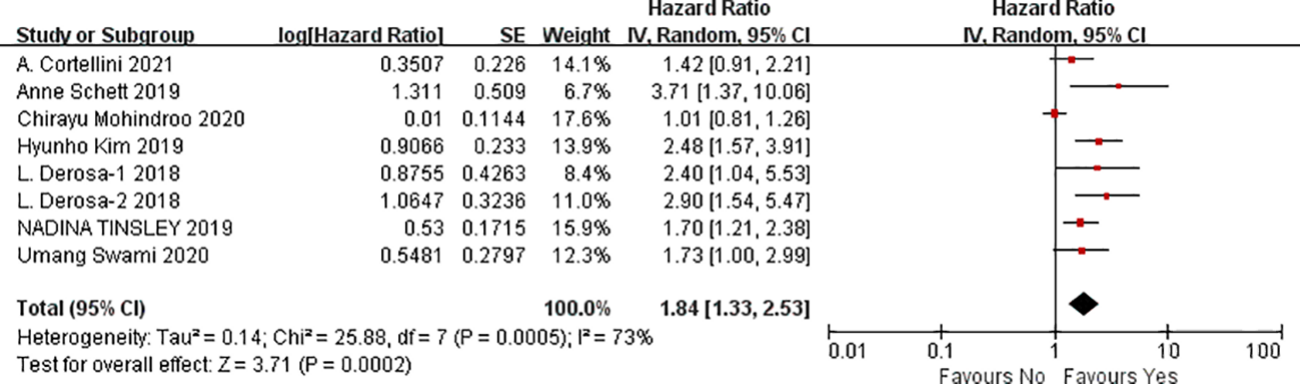
Meta-analysis results of antibiotics (within the previous 2 months) in overall survival.

A total of seven studies were available to analyze the influencing factors of overall survival in cancer patients treated with ICIs under antibiotic exposure. The outcome indicators mainly included gender (male vs. female), age (<65 vs. ≥65), smoking status (smoking vs. non-smoking), ECOG score (1–2 vs. 0), previous cancer history (yes vs. no), brain metastasis (yes vs. no), liver metastasis (yes vs. no), previous systemic therapy (yes vs. no), radiation in the past 3 months, history of antibiotic use in the past 2 months, PD-L1 expression level (1%–49%, ≥50%), and combined PD-1 drugs (nivolumab, pembrolizumab, and atezolizumab). The results of the meta-analysis showed that, under the influence of these factors, including gender (male vs. female) (HR = 1.19, 95%CI = 1.01–1.39, *P* = 0.04), ECOG PS (1–2 vs. 0) (HR = 2.19, 95%CI = 1.72–2.79, *P* < 0.0001), history of another cancer (yes vs. no) (HR = 1.60, 95%CI = 1.27–2.01, *P* < 0.0001), brain metastasis (yes vs. no) (HR = 1.41, 95%CI = 1.10–1.80, *P* = 0.007), liver metastasis (yes vs. no) (HR = 1.75, 95%CI = 1.28–2.38, *P* = 0.0004), radiation (within the previous 3 months) (HR = 2.18, 95%CI=1.38–3.45, *P* = 0.0008), antibiotics (within the previous 2 months) (HR = 1.84, 95%CI = 1.33–2.53, *P* = 0.0002), PD-L1 (1%–49%) (HR = 1.08, 95%CI = 1.03–1.13, *P* = 0.0008), and PD-L1 (≥50%) (HR = 1.78, 95%CI = 1.20-2.63, *P* = 0.004). The treatment of tumor patients with immune checkpoint inhibitors under antibiotic exposure led to a significant reduction in overall survival, and the difference was statistically significant. However, the use of antibiotics during ICI treatment was more significant in cancer patients with ECOG PS1–2 scores and radiation (within the previous 3 months), with 2.19- and 2.18-fold reductions in OS. Next, antibiotics (within the previous 2 months), PD-L1 (≥50%) and liver metastasis had a greater impact on the shortened overall survival of patients treated with antibiotics during immune checkpoint inhibitors which was 1.84, 1.78, and 1.75 times of those without antibiotic treatment, respectively. In clinical treatment, such patients should be screened, and their medical history should be inquired in detail to avoid the use of antibiotics in immunotherapy, which may lead to shortened OS and poor prognosis. However, age (<65 vs. ≥65), smoking status (smoking vs. no smoking), previous systemic treatment (yes vs. no), and combined PD-1 inhibitors (nivolumab, pembrolizumab, and atezolizumab) were not associated with OS, and the results were not statistically significant, as shown in **Figures 6**–**8** (due to the large number of figures, only the top three influencing factors are reflected) and **Table 4**.

**Table 4:** Meta-analysis results of the predictors of overall survival.

Outcomes	Studies	Heterogeneity test results		Effect model	Meta-analysis results	
		*P*	*I^2^*		95%CI	*P*
Gender (male vs. female)	5	0.77	0%	Fixed	1.19 [1.01, 1.39]	0.04
Age (<65 vs. ≥65)	6	0.65	0%	Fixed	1.01 [1.00, 1.03]	0.05
Smoking status (smoking vs. non-smoking)	4	0.70	0%	Fixed	1.51 [1.00, 2.27]	0.05
ECOG PS (1–2 vs. 0)	5	0.20	33%	Fixed	2.19 [1.72, 2.79]	< 0.0001
History of another cancer (yes vs. no)	6	0.24	26%	Fixed	1.60 [1.27, 2.01]	< 0.0001
Brain metastasis (yes vs. no)	5	0.13	43%	Fixed	1.41 [1.10, 1.80]	0.007
Liver metastasis (yes vs. no)	2	0.28	15%	Fixed	1.75 [1.28, 2.38]	0.0004
Prior systemic therapy (yes vs. no)	3	0.15	47%	Fixed	1.26 [0.98, 1.61]	0.07
Radiation (within the previous 3 months)	2	0.52	0%	Fixed	2.18 [1.38, 3.45]	0.0008
Antibiotics (within the previous 2 months)	8	0.0005	73%	Random	1. 84 [1.33, 2.53]	0.0002
PD-L1 (1%–49%)	3	0.58	0%	Fixed	1.08 [1.03, 1.13]	0.0008
PD-L1 (≥50%)	3	0.44	0%	Fixed	1.78 [1.20, 2.63]	0.004
Anti-PD(L)1 compound—nivolumab	2	0.001	91%	Random	1.00 [1.00, 1.00]	0.36
Anti-PD(L)1 compound—pembrolizumab	2	0.35	0%	Fixed	1.48 [0.96, 2.29]	0.08
Anti-PD(L)1 compound—atezolizumab	2	0.90	0%	Fixed	1.23 [0.65, 2.32]	0.52

ECOG PS, Eastern Cooperative Oncology Group performance status.

### Publication bias test

**Figure 9:**
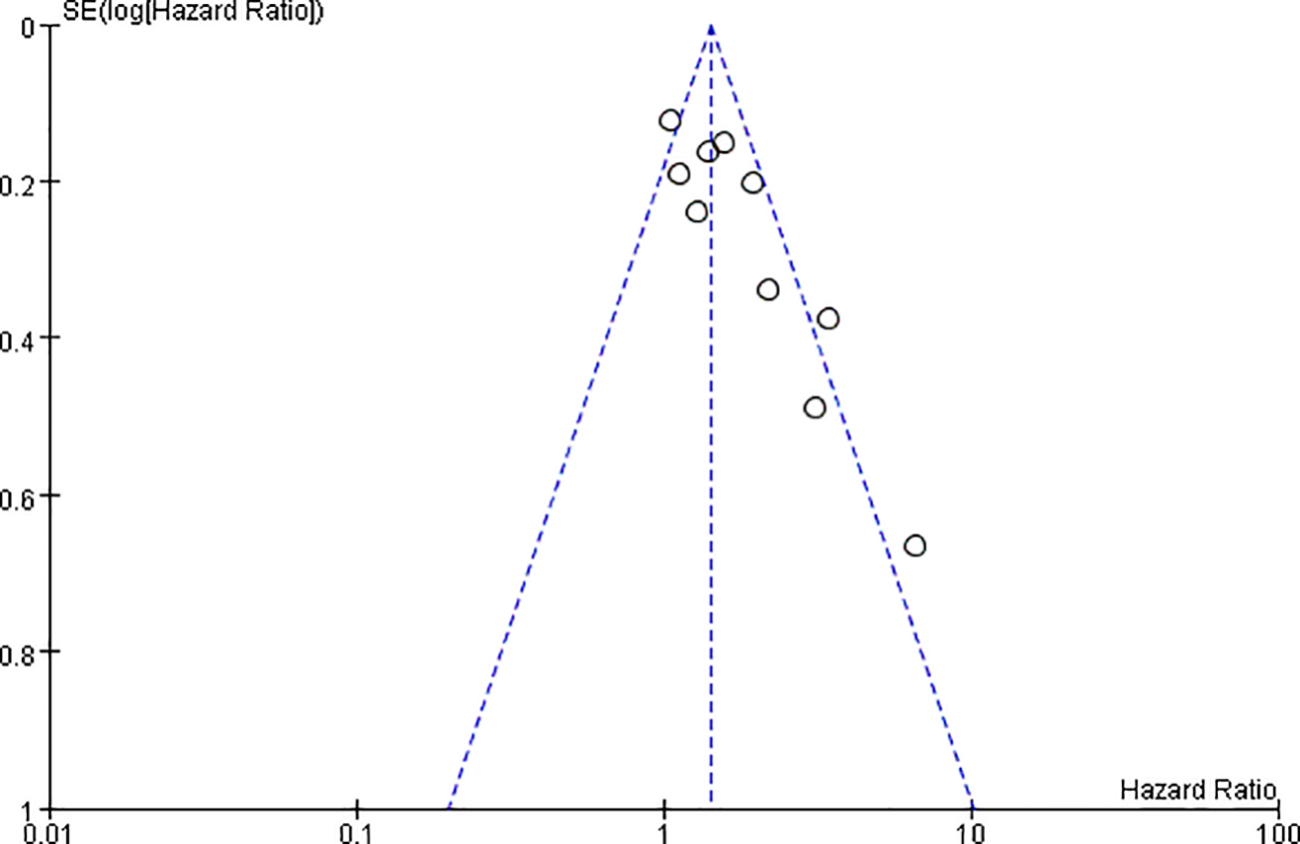
Publication bias funnel plot of progression-free survival in patients with antibiotics (within the previous 2 months).

The publication bias of this study was evaluated based on the inverted funnel plot of the PFS of patients with antibiotics (within the previous 2 months). The funnel plot was drawn with HR as the abscordinate and logHR as the ordinate. The results showed that the scatter points of the included studies were relatively concentrated in general. No significant asymmetry was shown, indicating that the publication bias of the included studies was small, as shown in **Figure 9**.

## Discussion

Cancer patients should be given a reasonable treatment plan, and the treatment plan should be designed and screened. Immune checkpoint inhibitors have become one of the important and effective treatments for many cancers. Clinical studies in first-line treatment have shown that immune checkpoint inhibitors can significantly prolong the PFS and OS of tumor patients (20–23). The development, progression, and metastasis of tumors are often accompanied by a systemic inflammatory response, and antibiotics are often used in this process. Antibiotics can cause intestinal flora imbalance, which may be one of the reasons affecting the effect of immune checkpoint inhibitors (24). The study showed that the antitumor effect and survival of tumor-bearing mice treated with PD-1 inhibitor after 14 days of broad-spectrum antibiotics in a sterile environment were significantly worse than those of tumor-bearing mice raised in a common environment. Fecal microbiota from cancer patients who were effective to PD-1 inhibitor treatment restored the antitumor effect of PD-1 inhibitor when transplanted into the intestinal tract of the former mice, but no significant antitumor effect was found when fecal microbiota from cancer patients who were ineffective to PD-1 inhibitor was transplanted into the former mice (25). Another clinical study (9) showed that patients who received antibiotics within 2 months before receiving immune checkpoint inhibitors had a significantly shorter PFS than those who did not (median PFS 1.4 vs. 5.5 months, HR = 2.22, *P* < 0.01). It can be speculated that antibiotics affect the clinical benefit of immune checkpoint inhibitors by changing the state of the microbial flora. However, it remains questionable whether good biomarkers are reliable enough to guide immunotherapy in patients with more severe infections and in whom antibiotics must be administered. Assessing reliable predictors of immune checkpoint inhibitor response to guide clinicians in the selection of patients who are likely to benefit from these agents in the immunotherapy era remains a challenge. In this study, we analyzed 16 clinical and molecular features to evaluate the clinical prognosis of cancer patients receiving immune checkpoint inhibitor therapy under antibiotic exposure to guide the clinical decision-making.

We analyzed the effects of age (<65 vs. ≥65), gender (male vs. female), ECOG PS (1–2 vs. 0), smoking status (smoking vs. non-smoking), PD-L1 expression level (1%–49%, ≥50%), previous cancer history (yes vs. no), brain metastasis (yes vs. no), liver metastasis (yes vs. no), radiation (within the previous 3 months), BRAF mutation status (yes vs. no), antibiotic use history (within the previous 2 months), and co-use of PD-1 inhibitors on the PFS and OS of tumors treated with immune checkpoint inhibitors under antibiotic exposure.

In terms of PFS, gender, ECOG PS, history of another cancer, liver metastasis, antibiotics (within the previous 2 months), PD-L1 (1%–49%), and PD-L1 (≥50%) had a significant impact on the progression-free survival of tumors treated with immune checkpoint inhibitors under antibiotic exposure. Among them, the PD-L1 expression level >50% in cancer patients shortened the PFS most severely, and the PD-L1 expression level of 1%–49% had almost no effect on PFS. Therefore, it is important to evaluate the practicality of PD-L1 detection in real-world practice and to identify reliable predictors of adverse clinical effects of antibiotic use in patients receiving immunotherapy. Moreover, there was also a significant effect of ECOG PS (1–2 vs. 0) on progression-free survival of tumors treated with immune checkpoint inhibitors under antibiotic exposure. The ECOG score is an indicator of a patient’s general health and tolerance to treatment of their physical strength. Lower scores indicate better patient performance status. Our results showed that patients with an ECOG PS score of 1–2 had a significantly worse prognosis when treated with immune checkpoint inhibitors under antibiotic exposure compared with patients with an ECOG PS score of 0. In addition, the history of other cancers and the use of antibiotics in the past 2 months also have a negative impact on the PFS of patients. In addition, gender, liver metastasis, and PD-L1 (1%–49%) factors also had small effects on the progression-free survival of tumors treated with immune checkpoint inhibitors under antibiotic exposure, and the results were statistically significant. Among other factors, age (<65 vs. ≥65), smoking status (smoking vs. non-smoking), brain metastasis (yes vs. no), BRAF mutation (yes vs. no), prior systemic therapy (yes vs. no), radiation (within the previous 3 months), and anti-PD(L)1-compound—nivolumab/pembrolizumab/atezolizumab were not statistically significant. In summary, for patients without disease progression, PD-L1 (≥50%), ECOG PS1–2 score, history of another cancer (yes vs. no), and antibiotics (within the previous 2 months) were reliable predictors of the adverse clinical effect of antibiotics in patients receiving immunotherapy. Antibiotics should be avoided in these cases.

In terms of OS, gender, ECOG PS (1–2 vs. 0), history of another cancer, brain metastasis, liver metastasis, radiation (within the previous 3 months), antibiotics (within the previous 2 months), PD-L1 (1%–49%), and PD-L1 (≥50%) had a significant impact on overall survival in tumors treated with immune checkpoint inhibitors under antibiotic exposure. Among them, ECOG PS of 1–2 and radiation (within the previous 3 months) shortened OS most significantly, followed by antibiotics (within the previous 2 months), PD-L1 (≥50%), liver metastasis (yes vs. no), and history of another cancer (yes vs. no). Gender, brain metastasis (yes vs. no), and PD-L1 (1%–49%) also had a small impact on the overall survival of tumors treated with immune checkpoint inhibitors under antibiotic exposure, and the results were statistically significant. Other factors, for example, age (<65 vs. ≥65), smoking status (smoking vs. non-smoking), prior systemic therapy, and anti-PD(L)1 compound—nivolumab/pembrolizumab/atezolizumab, were not statistically significant. In conclusion, clinicians can evaluate the patient’s status and use antibiotics rationally based on the above-mentioned factors.

This study was essentially a meta-analysis of nine randomized controlled clinical trials based on published literature. Although we collected enough information, there were still some limitations. Many important details of the included studies, such as tumor type, limited our further analysis to a certain extent and affected our results. In addition, the potential publication bias in this study, although it did not significantly affect the conclusions, is that it still needs to include more studies in subsequent studies for improvement. However, it is undisputed that the above-mentioned related predictors can provide more accurate information on the adverse clinical effect of antibiotics in patients receiving immunotherapy, which shows great potential in clinical application and will provide strong support for clinicians to use antibiotics rationally.

## Data availability statement

The original contributions presented in the study are included in the article/supplementary material. Further inquiries can be directed to the corresponding authors.

## Author contributions

We declare that all authors contributed greatly to the manuscript. ZZ and XL searched the database and analyzed the data. QC and SF selected the study and extracted the data. QC and SL wrote the manuscript. SF and SL reviewed the manuscript. All authors contributed to the article and approved the submitted version.
